# Body-shape trajectories and their genetic variance component in Gilthead seabream (*Sparus aurata* L.)

**DOI:** 10.1038/s41598-021-95726-9

**Published:** 2021-08-20

**Authors:** Stefanos Fragkoulis, Dimosthenis Kerasovitis, Costas Batargias, George Koumoundouros

**Affiliations:** 1 Biology Department, University of Crete, Vasilika Vouton, 70013 Heraklion, Crete, Greece; 2 Avramar S.A, PEO Patron-Athinon 55, Agios Vasilios, 26500 Rion, Greece; 3 Animal Production, Fisheries and Aquaculture, University of Patras, Nea Ktiria, 30200 Messolonghi, Greece

**Keywords:** Marine biology, Ichthyology

## Abstract

The phenotype of juvenile fish is closely associated with the adult phenotype, thus consisting an important quality trait for reared fish stocks. In this study, we estimated the correlation between the juvenile and adult body-shape in Gilthead seabream, and examined the genetic basis of the ontogenetic trajectories. The body shape of 959 pit-tagged fish was periodically examined during the juvenile-to-adult period. Individual shape ontogenetic trajectories were studied in respect to the initial (juvenile) and final (adult) phenotypes, as well as to the rate that adult phenotype is attained (phenotypic integration rate). We found that the juvenile body-shape presented a rapid change up to 192.7 ± 1.9 mm standard length, followed by a phenotypically stable period (plateau). Depending on the shape component considered, body-shape correlations between juvenile and adult stages ranged from 0.22 to 0.76. Heritability estimates (h^2^) of the final phenotype ranged from 0.370 ± 0.077 to 0.511 ± 0.089, whereas h^2^ for the phenotypic integration rate was 0.173 ± 0.062. To our knowledge, this is the first study demonstrating that the variance of the ontogenetic trajectories has a substantial additive genetic component. Results are discussed in respect to their potential use in selective breeding programs of Gilthead seabream.

## Introduction

Body-shape is a significant quality trait of reared fish, especially in the case of species that are marketed as a whole^1–4^. Except of its normal intra-species variation, under rearing conditions, body-shape is frequently subjected to the effects of skeletal abnormalities, which develop mostly during the hatchery phase^5^. Reasonably, juvenile phenotype has been considered as a good predictor of fish morphological quality at harvesting, with commercial hatcheries adopting certain practices to sort out the abnormal individuals, before they are transferred in sea cages^5,6^. After their transfer in cage farms, normal juveniles have rarely recorded to develop severe skeletal abnormalities^7^, or to recover from an existing skeletal abnormality^8^.

As every phenotypic trait, fish body-shape is determined by the action of both the environment and genotype. Abiotic parameters and nutrition can exert a direct effect on fish body-shape at given developmental periods^9–12^, or modify the ontogenetic rate of body-shape^13–15^, without necessarily leading to long-lasting phenotypic changes. In a similar way, genotype has been proven as a significant source of body-shape variation in reared fish, with the majority of existing studies targeting the near harvesting stages to improve the final product^1,2,4,16–18^.

In most finfish species, body-shape ontogeny does not follow a uniform pattern, but presents different periods of rapid allometric changes, usually ending to a period of rather isometric growth^19–21^. Despite our interest in predicting the final phenotype of reared fish, to our knowledge, no studies exist on the correlation of fish body-shape between different developmental stages, at the individual level. Relevant literature on other organisms (e.g., humans) demonstrates significant phenotypic correlations between different ontogenetic stages and a substantial variability in individual body-shape trajectories^22^. Furthermore, other studies show that the ontogenetic trajectories of various phenotypic traits (pigmentation pattern in snakes, herbivore defensive traits in plants, timing of developmental events in pond snails) may be heritable^23–25^.

In the present study, we examined whether juvenile body-shape is a good predictor of the adult body-shape in Gilthead seabream (*Sparus aurata* L.). Individual ontogenetic trajectories of body-shape were quantified for 959 fish and their genetic parameters were estimated. The studied organism is an important species of European aquaculture, which is marketed primarily as a whole. In the past, clear quality criteria have been expressed by consumers with respect to the body-shape of reared Gilthead seabream^3^.

## Results

### Correlation of body-shape between different sampling ages

**Figure 1:**
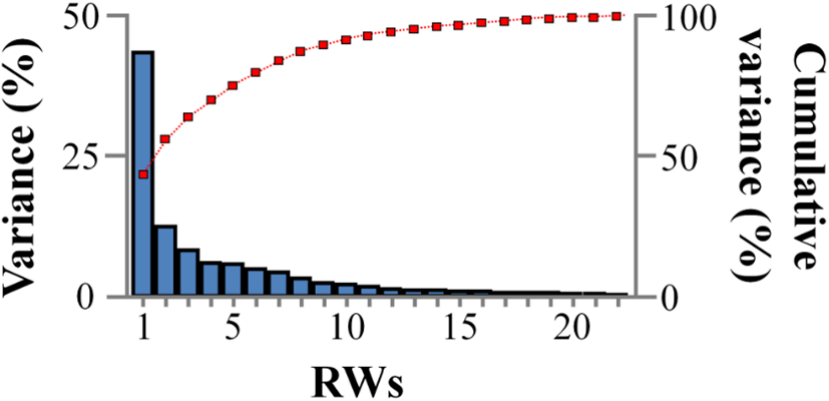
Variance explained by the twenty-two relative warps (RWs).

**Figure 2:**
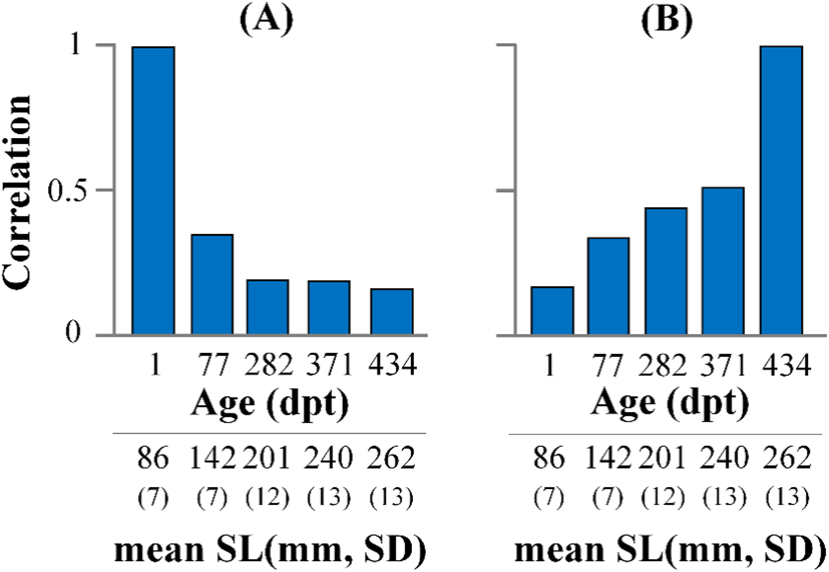
(**A**) Pearson’s correlation coefficients between the Procrustes distances of the juvenile stage (1 dpt) with the Procrustes distances of the next sampling points, up to the end of the on-growing period (434 dpt). (**B**) Pearson’s correlation coefficients between the Procrustes distances at the end of on-growing (434 dpt) with the Procrustes distances of the previous sampling points, up to the beginning of on-growing period (1 dpt). The average standard length (SL) of each sample is given. All estimated correlations were significant (*P* < 0.05).

Relative warp analysis on all the specimens and sampling ages produced twenty-two relative warps (RWs), with RW1 explaining the 43.4% of total variance (Fig. 1). To quantify the overall body-shape change during ontogeny, all the estimated RWs were used to calculate Procrustes distances (PDs) of each fish at the different sampling ages. Juvenile body shape was significantly correlated with that of the following sampling ages, with the correlation coefficient decreasing with fish growth, from 0.34 between the body shape at 1 and 77 dpt (PD_1_-PD_77_), to 0.16 between body shape at 1 and 434 dpt (PD_1_-PD_434_, Fig. 2A). Similarly, fish body shape at the end of on-growing (434 dpt) was significantly correlated with the body shape of earlier samples, with the correlation coefficient decreasing as the age difference between the samples increased (r = 0.51 for PD_434_-PD_371_, to r = 0.16 for PD_434_-PD_1_, Fig. 2B).

**Figure 3:**
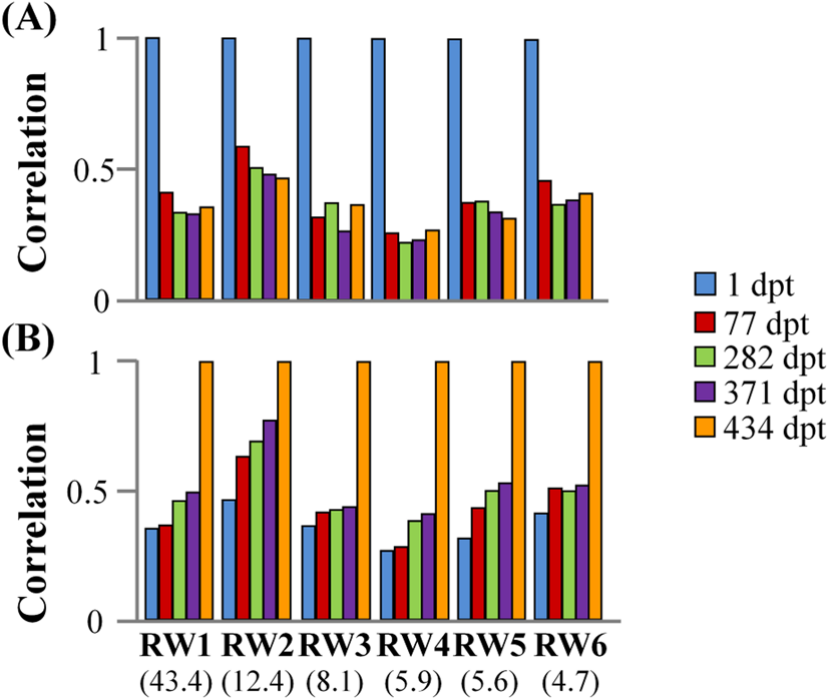
(**A**) Pearson’s correlation coefficients between the relative warps (RW) of the juvenile stage (1 dpt) with the RWs of the next sampling points, up to the end of the on-growing period (434 dpt). (**B**) Pearson’s correlation coefficients between the relative warps (RW) at the end of on-growing (434 dpt) with the RWs of the previous sampling points, up to the beginning of on-growing period (1 dpt). Correlation coefficients were estimated for the first six relative warp scores which explain the 80.1% of total body-shape variance. Numbers in brackets give the variance explained by each RW. All estimated correlations were significant (*P* < 0.05).

To examine whether different shape components show different correlations between the different sampling ages, we examined the correlation of individual RW’s between the first (1 dpt) or the last sample (434 dpt, from now on "reference-age-samples") with the rest samples. Analysis included the first six relative warps (RW_1_-RW_6_) which cumulatively explained the 80.1% of the total variance (Fig. 1). For all the examined RW’s, a significant correlation (p < 0.05) was found between the reference-age-samples and the rest samples. Similarly, to what was observed in PDs, in most of the cases, the correlation values decreased as the age difference between samples was increased (Fig. 3). Depending on the RW under concern, correlation coefficients ranged from 0.22 to 0.76. Interestingly, the higher correlation values were observed in RW2, for both reference-age-samples examined (0.46 to 0.58 and 0.45–0.76 for the 1 and 434 dpt samples respectively, Fig. 3).

### Body shape ontogeny during the on-growing period

**Figure 4:**
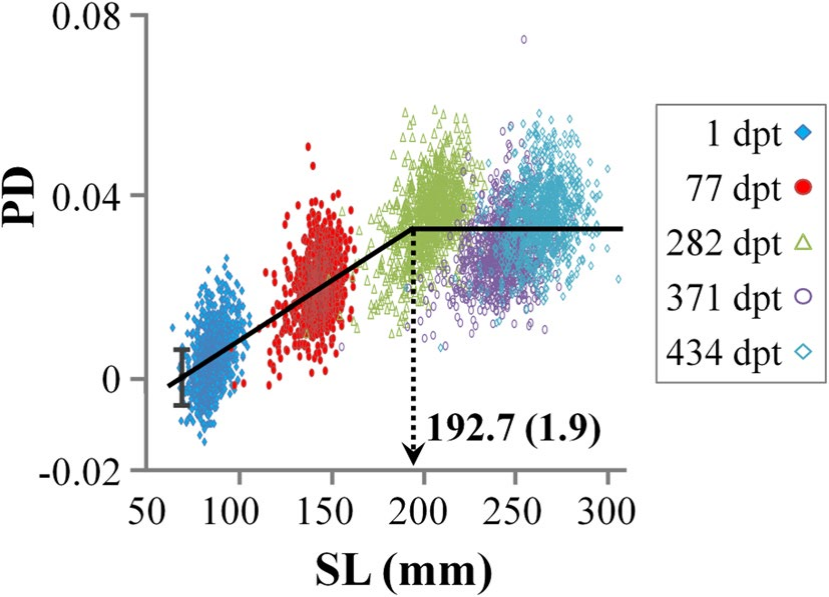
Relationship of the Procrustes distances (PD) to standard length (SL) throughout the on-growing period. Error bars (SD) correspond to the mean Procrustes distance of the 30 juveniles with the smallest SL, which composed the reference for the calculation of PDs.

**Figure 5:**
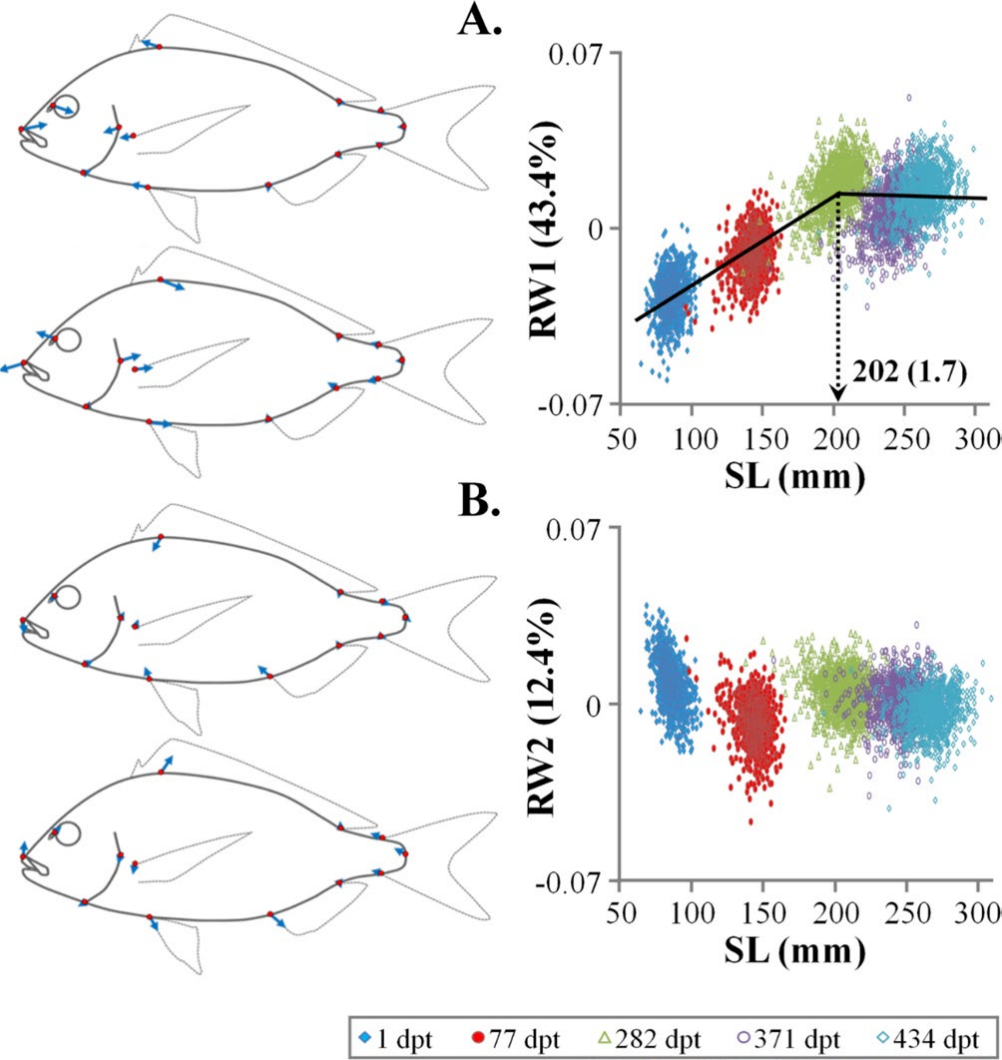
Relationship of (**A**) the first relative warp (RW1) and (**B**) the second relative warp (RW2) to standard length (SL) throughout the on-growing period. Vector diagrams demonstrate the components of shape change relative to the extreme values of Y-axes (on the observed scale).

As it was demonstrated by the graph of Procrustes distances on SL, there was a significant size-effect on seabream body shape during the on-growing period (Fig. 4). In the SL range between ca 70 and 192.7 ± 1.9 (± SE) mm SL the PDs increased with the growth of the fish, whereas in the following ontogenetic period it was independent of SL (Fig. 4, Table S1). Following relative warp analysis, size-effects on body shape were evident for RW1 (43.4% of the total variance explained, Fig. 5A). Simlarly to what was observed for PD, RW1 changed with fish growth up to 202.0 ± 1.7 (± SE) mm SL, whereas over this size RW1 was independent of SL (Fig. 5A). Ontogenetic shape variation between juvenile and adult samples mainly concerned the anterior body parts. The transition from the juvenile to adult phenotype was characterised by posterior shift of the snout and the anterior margin of the eye, as well as by an anterior transposition of the gill-cover, pelvic and pectoral-fin bases (Fig. 5A).

In opposite to RW1, no abrupt changes during fish growth were observed in the case of RW2 (12.4% of the total variance explained, Fig. 5B). Shape variation across RW2 axis mainly concerned the proximal shift of the dorsal profile (landmark 13), of the pectoral-fin bases (landmark 8) and of the anterior anal-fin base (landmark 7), as well as the ventral shift of the snoot (Fig. 5B). Similarly, to RW2, no size effects were evident in the case of the rest four relative warps (RW3-RW6, Fig. S1) that cumulatively explained 24.3% of the total body-shape variance.

### Quantification of body-shape trajectories

**Figure 6:**
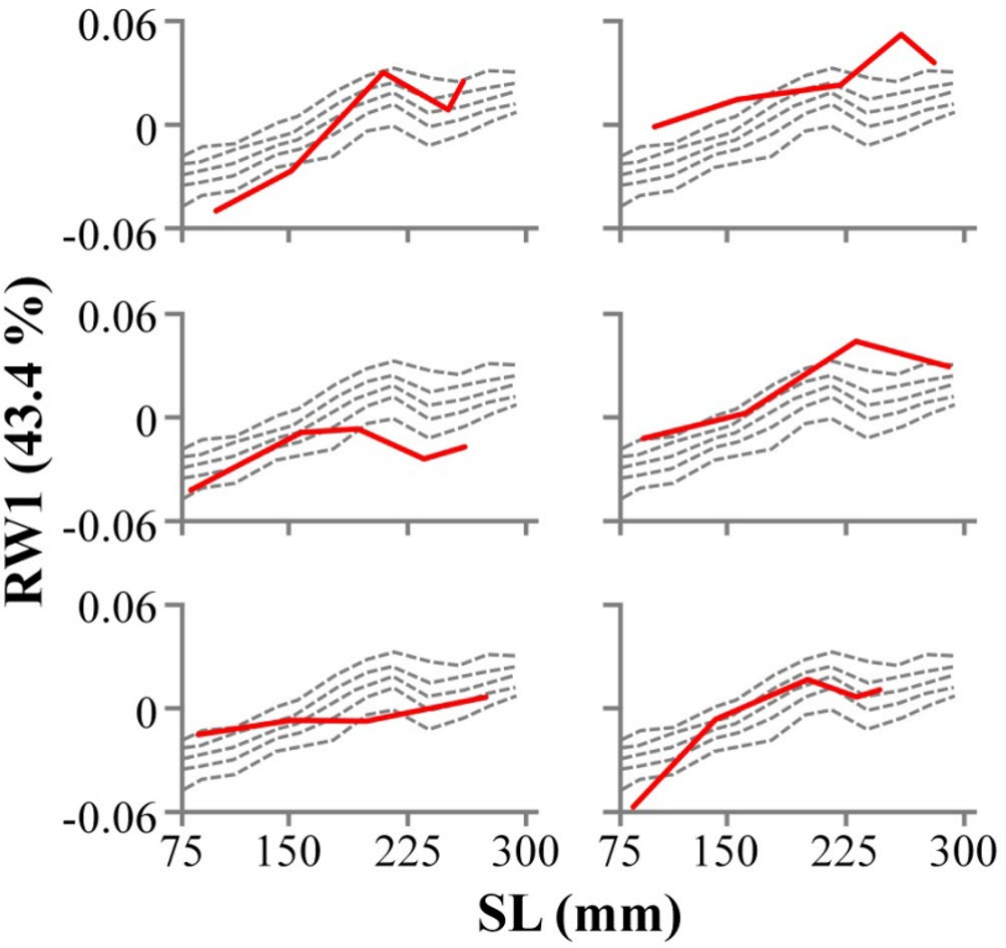
Examples of individual body-shape (RW1) trajectories (solid lines) in relation to the consensus population trajectory (dashed lines), as it is defined by the 5 (lower dashed line), 25, 50, 75 and 95 (upper dashed line) percentiles.

To visualize the body-shape trajectory of each individual in respect to the consensus trajectory, shape data were categorized into twelve SL classes of 20 mm each (Table S2). Consensus allometric trajectories were plotted on the 5, 25, 50, 75 and 95 percentiles of each SL class (Fig. 6, Table S2). Interestingly, different body-shape (RW1) trajectories could be observed in different individual fish (Fig. 6).

To quantify the body-shape trajectory which was followed by each individual fish, the linear-regression parameters (slope, intercept) of SL-RW1 and SL-PD relationships were estimated separately for each specimen during the period of the first three samples (phase 1); i.e., when body-shape changes rapidly with the growth of SL (1–282 dpt, Fig. 6). Estimated coefficients of determination (r^2^) were high for all the fish, both in the case of RW1 (0.34–1) and PD (0.13–1) (Table S3). For the period of the last three samples (phase 2), when RW1 and PD were not affected by SL (282–434 dpt, Fig. 6), the mean RW1 and PD were calculated separately for each individual, as a body-shape estimate at the trajectory plateau (pL-RW1, pL-PD, Table S3). Following the lack of abrupt changes of RW2 with SL growth (Fig. 5B), the mean RW2 of each individual (av-RW2) was calculated to describe the plateau of SL-RW2 trajectory throughout the entire studied period (1–434 dpt).

### Genotyping and parental assignment

From the 959 animals that were used to collect data, 913 (95%) were assigned to either one single pair or one single parent eligible to be used to quantitative further analysis. From these, the 84% of the offspring was assigned to a single pair whereas 11% to one parent but multiple pairs. The sires that participated to the spawning were 59 out of 60 whereas the dams were 54 out of 59. The number of full-sib families formed was 374, whereas the number of maternal and paternal half-sib families was 54 and 59 respectively. The full-sib family size ranged from 1 to 21 with an average family size of 2.2 and family size variance of 4.84. The paternal half-sib family size ranged from 1 to 43 with an average family size of 12.7 and family size variance of 8.9, whereas the maternal half-sib family size ranged from 1 to 72 with an average family size of 15 and variance of 13.8.

### Genetic parameters of body-shape trajectories

Heritability estimates of all PD-SL trajectory parameters were statistically significant, ranging from 0.098 (Y-intercept, a-PD), to 0.173 (slope, b-PD) and 0.390 (plateau, pL-PD) (Table 1). Similar results were observed for the RW1-SL trajectory parameters, with however insignificant h^2^ estimate for the slope (b-RW1) (Table 1). A high heritability estimate was present for av-RW2 (0.511, Table 1). Finally, h^2^ estimates for fish standard length (SL) increased with fish age, ranging from 0.159 (1 dpt) to 0.291 (434 dpt, Table 1).

**Table 1:** Estimates of heritabilities (h^2^) for body-shape trajectory traits (RW1 and PD) and fish standard length (SL).

Trajectory	Parameter	Abbreviation	h^2^ ± SE	*P*
SL-PD	Slope (1–282 dpt)	b-PD	0.173 ± 0.062	*P* < 0.05
	Plateau (282–434 dpt)^2^	pL-PD	0.390 ± 0.078	*P* < 0.01
	Y-intercept	a-PD	0.098 ± 0.048	*P* < 0.05
SL-RW1	Slope (1–282 dpt)	b-RW1	0.024 ± 0.035	ns
	Plateau (282–434 dpt)^1^	pL-RW1	0.370 ± 0.077	*P* < 0.01
	Y-intercept	a-RW1	0.105 ± 0.049	*P* < 0.05
SL-RW2	Mean RW2 (1–434 dpt)	av-RW2	0.511 ± 0.089	*P* < 0.01
	SL (1 dpt)	SL_1_	0.159 ± 0.055	*P* < 0.01
	SL (77 dpt)	SL_77_	0.198 ± 0.063	*P* < 0.01
	SL (282 dpt)	SL_282_	0.261 ± 0.064	*P* < 0.01
	SL (371 dpt)	SL_371_	0.274 ± 0.067	*P* < 0.01
	SL (434 dpt)	SL_434_	0.291 ± 0.071	*P* < 0.01

*P* values indicate the significance of the estimates’ difference from zero, *ns* not significant difference, *dpt* days post-tagging, *PD* Procrustes distance, *RW*1, *RW*2 scores of the 1st and 2nd relative warp axis respectively.

1, mean RW1 (282–434 dpt).

2, mean PD (282–434 dpt).

Phenotypic and genetic correlations were estimated for all the examined traits, including those with insignificant heritabilities (Table 2). Generally, significant genotypic correlations were substantially fewer than the significant phenotypic. Negative high genetic (-0.717 to -0.800) and phenotypic (-0.897 to -0.905) correlations were found between the slope and Y-intercept of both SL-RW1 and SL-PD ontogenetic relationships examined. Phenotypic and genetic correlations between fish SL at different sampling ages increased as age difference decreased, ranging between 0.179–0.888 (phenotypic, r_p_) and 0.273–0.948 (genetic, r_g_). In the most of the rest trait pairs examined, phenotypic correlations were significant but low (Table 2). The significant genetic correlations between body-shape traits and SL were low, with a maximum value of 0.461 (pL-PD with SL_282_). No significant genetic correlations were observed between av-RW2 and SLs. Interestingly, significant genetic correlations found between av-RW2 and the plateau phenotypes of SL-PD (a-PD, 0.518) and SL-RW1 (a-RW1, -0.644) trajectories (Table 2). Concerning the rest trajectory parameters, a significant medium genetic correlation was observed between pl-PD and b-PD (0.672), as well as between pl-RW1 and a-RW1 (0.652) (Table 2).

**Table 2:** Phenotypic (above diagonal) and genetic (under diagonal) correlations among traits of body-shape trajectory traits (RW1, PD and RW2) and fish standard length (SL).

	b-PD	pL-PD	a-PD	b-RW1	pL-RW1	a-RW1	av-RW2	SL_1_	SL_77_	SL_282_	SL_371_	SL_434_
b-PD	***0.169***	**0.462**	**− 0.905**	**0.434**	**0.507**	**− 0.152**	**0.220**	**− 0.075**	**− 0.080**	**0.074**	**0.104**	0.058
pL-PD	**0.672**	***0.386***	**− 0.188**	**0.247**	**0.878**	0.006	**− 0.135**	**0.154**	**0.189**	**0.397**	**0.389**	**0.331**
a-PD	**− 0.800**	**− **0.172	***0.145***	**− 0.277**	**− 0.324**	**0.103**	**− 0.386**	**0.107**	0.062	**− **0.057	**− 0.106**	**− 0.093**
b-RW1	0.048	0.143	0.205	***0.051***	**0.229**	**− 0.897**	**− 0.116**	0.056	**− 0.144**	**− 0.077**	**− 0.078**	**− 0.125**
pL-RW1	**0.707**	**0.882**	**− **0.381	**− **0.083	***0.373***	**0.075**	**0.245**	**0.121**	**0.189**	**0.398**	**0.418**	**0.388**
a-RW1	0.486	**0.447**	**− **0.403	**− 0.717**	**0.652**	***0.134***	**0.278**	**− 0.119**	**0.071**	0.062	**0.082**	**0.124**
av-RW2	**− **0.475	0.223	**0.518**	0.310	**− **0.195	**− 0.644**	***0.508***	**− 0.240**	**− 0.139**	**− **0.040	0.051	**0.107**
SL_1_	0.203	0.102	**− **0.219	**− **0.295	0.072	0.397	0.012	***0.131***	**0.582**	**0.333**	**0.230**	**0.179**
SL_77_	**− **0.240	0.326	0.188	0.145	0.220	**− **0.135	0.140	**0.460**	***0.185***	**0.621**	**0.492**	**0.443**
SL_282_	**− **0.027	**0.461**	0.100	0.349	**0.425**	**− **0.290	0.110	**0.581**	**0.738**	***0.271***	**0.849**	**0.737**
SL_371_	**− **0.174	**0.407**	0.163	0.386	0.289	**− 0.456**	0.262	0.412	**0.682**	**0.948**	***0.280***	**0.888**
SL_434_	**− **0.346	0.311	0.221	0.288	0.226	**− **0.397	0.186	0.273	**0.571**	**0.854**	**0.943**	***0.297***

Diagonal values (italics) are the estimates of heritabilities of the traits under the multivariate model. Values in bold indicate statistically significant estimates (*P* < 0.05).

*b-PD* slope of Procrustes distances, *pL-PD* plateau of Procrustes distances, *a-PD* Y-intercept of Procrustes distances, *b-RW1* slope of the first relative warp, *pL-RW1* plateau of the first relative warp, *a-RW1* Y-intercept of the first relative warp, *av-RW2* mean value of the second relative warp between all age samples, *SLi* Standard length of fish for each sample.

## Discussion

The present study showed a significant but low correlation of body-shape between the juvenile and adult stage of Gilthead seabream. Body-shape ontogeny during the juvenile-to-adult period presented a substantial intra-population variation, in respect to the final (adult) phenotype (pl-PD), as well as the rate that this phenotype is attained (b-PD, slope or phenotypic integration rate). Significant heritability estimates for both of these traits (Table 1) showed that variation in shape ontogenetic trajectory has a significant genetic component. High heritability estimates for body-shape have been reported in a variety of fish species^1,26–28^. To our knowledge however, this is the first study documenting that variation in phenotypic integration rate has additive genetic component.

To follow the ontogeny of overall body-shape, in this study, Procrustes distances (PD) were estimated as an overall measure of multivariate shape components (i.e. relative warps, RWs). In agreement with previous studies^29^, PD successfully described the overall shape ontogeny with fish size (i.e., SL), additionally allowing the estimation of genetic parameters for body-shape trajectory. Compared with the relative warps however, PD showed smaller phenotypic correlation between juvenile and adult stage, and thus inferior capacity in predicting the adult from the juvenile body-shape. Relative warps analysis, analogous to Principal Components Analysis, replaces original shape variables with new ones (RWs) that are independent of each other, thus partitioning the overall shape variation into different components^30,31^. In the present study, significant differences were observed between the different RWs, not only in respect to the explained shape variation, but also in respect to their dependence on fish growth (allometry), correlation between the juvenile and adult stages and heritability estimates for final phenotypic scores (i.e. plateau phenotypes). Size-dependent shape variation (allometry) was expressed along the RW1 axis, whereas the rest RWs accounted mostly for size-independent shape variation. Such a partitioning is typical of relative warp analysis, especially when shape is studied over a relative wide size range^1,10,19,21^.

Body-shape correlations between different stages of fish ontogeny have been studied in the past, mostly with respect to the phenotypic evolution of abnormalities-related variation^32–34^. In Gilthead seabream, it was demonstrated that body shape is not altered after ca 70 mm total length, thus strengthening the hypothesis on the high predictability value of juvenile body shape^21^. In spite of their significance, in our study, shape correlations between different ages were rather low (0.22–0.76), even in the case of size-independent shape variables (i.e. RW2). Taking into account the environmental variation during fish growth in sea cages (e.g., water temperature^8^), this result suggests that seabream body-shape is subjected to the effects of genetics and environment throughout the entire juvenile-to-adult period. In agreement to this hypothesis, previous studies revealed that on-growing environment (i.e., tanks vs sea cages) has a significant effect on seabream body-shape at harvesting^3^. In agreement to other studies^21^, our results revealed a clear breakpoint during the ontogeny of Gilthead seabream body-shape, at however a substantially bigger fish length (193–202 mm SL). The difference in the estimated breakpoints between the two studies, might be attributed to the inclusion of larval period and to its effects on the overall shape trajectory^21^. The breakpoint observed in the present study might be associated with the process of sexual maturation, which in Gilthead seabream takes place after ca 20 cm TL^35^.

In the present study, moderate to high heritabilities (0.370–0.511) were estimated for Gilthead seabream shape-related traits at harvest (i.e. pl-PD, pl-RW1, av-RW2), clearly suggesting that body-shape can be exploited in a selective breeding program. Among the examined traits, interest should focus especially on RW2 because it explains a part of morphological variation (i.e., proximal/distal shift of the dorsal, anal and pelvic fins) which is related with the consumers’ preferences for the body-shape of reared Gilthead seabream^3^. This trait (RW2) has a high heritability (0.511) indicating that the accuracy and expected gain would be noticeable in a breeding program. Heritability of SL increases with age and this has been reported earlier for other species^36–38^ since the larger the distance from the early stages the less the impact of non-additive effects (genetic or environmental)^38,39^. On the contrary, the correlation estimates decrease with the age indicating that the distant ages have lower correlations, either genetic or phenotypic, since we are referring to different ontogenetic stages and developmental mechanisms. The RW2 is slightly correlated (either phenotypically or genetically) with the other shape traits (RW, PD and SLs) indicating that selection on one trait will slightly affect the other ones. The same stands for most of the genetic or phenotypic associations among SL, PD and av-RW traits. There are no reported correlations estimates for such trait associations (SL with PD or RW) in the literature. However, the reported genetic or/and phenotypic correlations of body weight with several traits of shape coincide very well with our estimates since they range between 0.2–0.5^1,2,4,16–18^.

During their ontogeny, animals follow rapid allometric changes to attain the adult phenotype. In organisms, like finfish, with external fertilization and planktonic early life stages, species-specific allometric growth patterns usually present multiple breakpoints between periods of different ontogenetic rates^21,40,41^. Environmentally-driven plasticity of ontogenetic trajectories may significantly alter the rate of shape ontogeny and the breakpoints (ontogenetic scaling), frequently leading to changes of body-shape at specific stages (i.e. juveniles, adults, etc.)^3,15,42^. Following the results of the present study, the rate of body-shape ontogeny is also subject to a genetically driven variation. In the future, it would be interesting to examine whether other organisms, besides fish, also present a strong genetic component with respect to their ontogenetic trajectory.

## Methods

### Fish origin and samplings

During this study no experimentation with alive animals was performed. The examined biological material consisted exclusively of digital photographs, which were derived from our previous study on lordosis recovery^8^. Examined fish group consisted of 959 seabream juveniles (86 ± 7 mm standard length, SL) with normal external morphology, which were tagged electronically (FDX-B, Trovan Ltd, USA) and examined periodically for their body-shape at tagging (1 dpt, days post-tagging, 155 dph, days post-hatching), and at 77 (232 dph), 282 (437 dph), 371 (526 dph) and 434 dpt (end of on-growing) (589 dph)^8^. During samplings, all specimens were anaesthetised (ethyleneglycol-monophenylether, Merck, 0.2–0.5 mL·L^−1^), scanned for ID recognition, photographed on the left side and returned to the sea-cage. The digital photographs of the specimen were taken with a Canon PowerShot G9 camera, mounted on a tripod^8^.

The examined fish population originated from a common larval population and egg batch, and reared according to the standard methodology for the species, in a land-based hatchery up to the juvenile stage and in a sea-cage farm for on-growing. The eggs were spontaneously spawned from a breeding population of 60 males and 59 females, which were kept under controlled photoperiod and temperature conditions. Fish rearing was performed at Andromeda S.A., a registered company (registration number GGN 5,200,700,699,992) for aquaculture production in Greece. The company is certified with GLOBAL G.A.P quality certification, with certified Veterinary doctor that verified the health and welfare of the fish. Animal sampling followed routine procedures and samples were collected by a qualified staff member from a standard production cycle. The legislation and measures implemented by the commercial producer complied with existing Greek (PD 56/2013) and EU (Directive 63/2010) legislation (protection of animals kept for farming). Production and sampling, by an experienced staff member, were optimised to avoid unnecessary pain, suffering or injury and to maximise fish survival.

### Morphometric analysis

**Figure 7:**
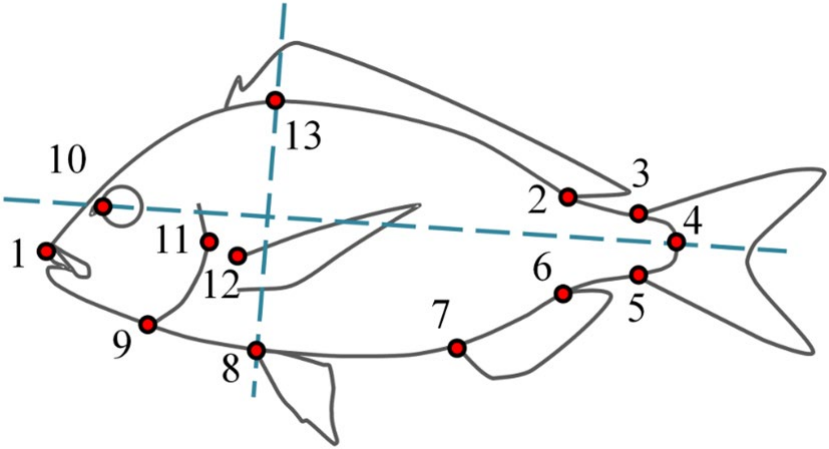
Landmarks collected in the present study. 1, Anterior tip of upper jaw; 2, posterior base of the dorsal fin; 3, dorsal tip of the base of caudal fin; 4, base of the central caudal lepidotrichium; 5, ventral tip of the base of caudal fin; 6, 7, posterior and anterior base of the anal fin, respectively; 8, base of the pelvic fins; 9, ventral tip of the gill cover; 10, anterior margin of the eye, dorsally to the nostril; 11, posterior tip of the gill cover; 12, dorsal base of the left pectoral fin; 13, projection of the landmark 8 on the dorsal profile of fish, perpendicularly to the axis which is defined by landmarks 4 and 10 (modified from Fragkoulis et al.^8^).

The ontogeny of body shape throughout the on-growing period was studied by geometric morphometry. On the digital photographs of the examined fish, thirteen homologous landmark measurements were taken by means of tpsDig2 software^43^ (Fig. 7). A generalized least square method was applied^44^ to adjust landmark configurations for centroid size and remove any irrelevant to shape variation. To compute the body-shape ontogenetic trajectories, the Thin-plate Spline algorithm was then applied and relative warp analysis (a principal component analysis of the partial warp scores^45^) was carried out on the resultant weight matrix (TpsRelw software^45^). To visualize the body-shape changes during the on-growing period, vector diagrams were obtained after the regression of shape components on the relative warp scores (tpsRegr software^46^).

As a proper metric for shape dissimilarity in the Kendall shape space, the Procrustes Distances (PDs) were used to estimate the overall body-shape changes of each specimen during growth^31,47^. For PD calculation, a subtotal of the thirty smaller juveniles (71.1 ± 2.1 mm SL, 1 dpt) was chosen as reference point. The Procrustes distance (PD) of each individual from the reference point was calculated by the formula:

$$PD = \sqrt {\sum \left( {RWi - rRWi} \right)^{2} } ,$$

where RWi is the individual score for each one of the estimated relative warps (RW1-RW22) and rRWi is the mean RWi for the reference group of fish. To examine whether the juvenile body-shape is a good predictor of fish body-shape at later stages, Pearson’s correlation coefficient between the individual PD values of the first (1 dpt) and each one of the next four sampling points (77, 282, 371 and 434 dpt) was estimated. This analysis was also repeated for the RW scores, each of which explained a specific part of body shape variation.

The SL at which body shape trajectories presented a breakpoint was estimated by a piecewise linear regression (software SPSS was used^48^), fitted with a non-linear estimation procedure

$${\text{Y}} = {\text{b}}_{0} + {\text{b}}_{{1}} \cdot {\text{SL}} + {\text{b}}_{{2}} \cdot \left( {{\text{SL}} - {\text{L}}} \right) \cdot \left( {{\text{SL}} \ge {\text{L}}} \right),$$

where Y is the studied body-shape variable (PD or RWs), SL is the standard length, b_0_ is the y-intercept, b_1_ is the slope of the Y-SL relationship during the first trajectory phase, b_2_ is the change in b_1_ that results in the slope of Y–SL relationship at the second trajectory phase, and L is the SL at the breakpoint^49^. To quantify the body-shape trajectory which was followed by each individual fish, the linear-regression parameters (slope, intercept) of the SL to shape-variables were estimated separately for each specimen and trajectory phase.

### Genotyping and parentage assignment

DNA of the breeders was extracted with Nucleospin 96 Tissue kit (Machery-Nagel, Germany), according to the manufacturer. DNA of the offspring was extracted using the Chelex-100 resin^50^. According to that, the tissue sample (of approximately 1 mm^2^) was added to a 96-well plate and mixed with 100 μl of 10% Chelex-100 resin solution and 15 μl of proteinase K (10 mg/ml, Boehringer Mannheim). The plate was then incubated at 55 °C for one hour (shaken from time to time) followed by a 30-min incubation at 100 °C to deactivate the proteinase K and extent of protein denaturation. The DNA extracts were stored at 4 °C or at -20 °C. Before every use, the mixture was vortexed and centrifuged at 10,000 rpm for 10 min to separate the surface layer in which the DNA can be found and the lower layer which contains the Chelex- 100 resin, the denatured proteins and other elements. 1–2 μl of supernatant are used for each 10 μl of final volume of the PCR reaction mixture.

All parents and offspring were genotyped using a multiplex-PCR of nine microsatellite markers^51–54^. 10 μl volumes containing 0.4 unit of Taq polymerase (KAPA Biosystems), 1 × Taq buffer, 0.2 mM dNTPs mix, 1.5 mM MgCl_2_, 0.35 μM of forward and 5′-fluorescently labelled reverse primer and approximately 20 ng of template DNA were used to perform the multiplex PCRs. An initial three-minute 95 °C denaturation step was followed by 34 cycles of 30 s at 95 °C, 30 s at 53 °C and 30 s at 72 °C, with a final extension at 72 °C for 20 min. An ABI PRISM 3500 DNA Analyzer (Applied Biosystems), was used to separate fluorescently labeled PCR products, with a GeneScan 500 LIZ Size Standard (ABI) internal size standard. GeneMapper (Applied Biosystems) software was used to size alleles and genotype individuals. The presence of genotyping errors or null alleles was checked with the software Micro-Checker^55^. Parentage assignment was performed by VITASSIGN software allowing two mismatch allele^56^.

### Data analysis

Heritabilities and phenotypic correlations were calculated using phenotypic data collected on 911 animals. Heritabilities were analyzed for all data using WOMBAT^57^. An animal model was fitted:

$$Y = {\text{X}}\beta + {\text{Z}}u + {\text{e}}\;\left( {{\text{model}}\;{1}} \right)$$

where Y is the vector of observations, β is the vector of fixed effects (overall mean), u is the vector of random additive genetic effects and e is the vector of random residual effects. X, Z are known incidence matrices. The genetic correlations were estimated for the traits applying a multivariate model.

### Ethical statement

During this study no experimentation with alive animals was performed. The examined biological material consisted exclusively of digital photographs, which were derived from our previous study on lordosis recovery^8^.

## Supplementary Information

Supplementary Information.

## Data Availability

The data that support the findings of this study are available from the corresponding author upon reasonable request.
