# Supplementary material for: Body-shape trajectories and their genetic variance component in Gilthead seabream (Sparus aurata L.)
Source: Sci Rep. 2021 Aug 20;11:16964. doi: 10.1038/s41598-021-95726-9 (PMC8379248; doi:10.1038/s41598-021-95726-9)
Supplement: Supplementary file 1 — Supplementary Information. [file 41598_2021_95726_MOESM1_ESM.docx]

Body-shape trajectories and their genetic variance component in Gilthead seabream (*Sparus aurata*, L)

Stefanos Fragkoulis^1^, Dimosthenis Kerasovitis^2^, Costas Batargias^3*^, George Koumoundouros^1*^

^1^ Biology Department, University of Crete, Vasilika Vouton, 70013 Heraklion, Crete, Greece, tel. +30 2810394065, Fax. +30-2810394408, [gkoumound@uoc.gr](mailto:gkoumound@uoc.gr)

^2^ Avramar S.A., PEO Patron-Athinon 55, Agios Vasilios, 26500 Rion, Greece

^3^ Animal Production, Fisheries and Aquaculture, University of Patras, Nea Ktiria, 30200 Messolonghi, Greece, [cbatargias@upatras.gr](mailto:cbatargias@upatras.gr)

*, to whom correspondence should be addressed


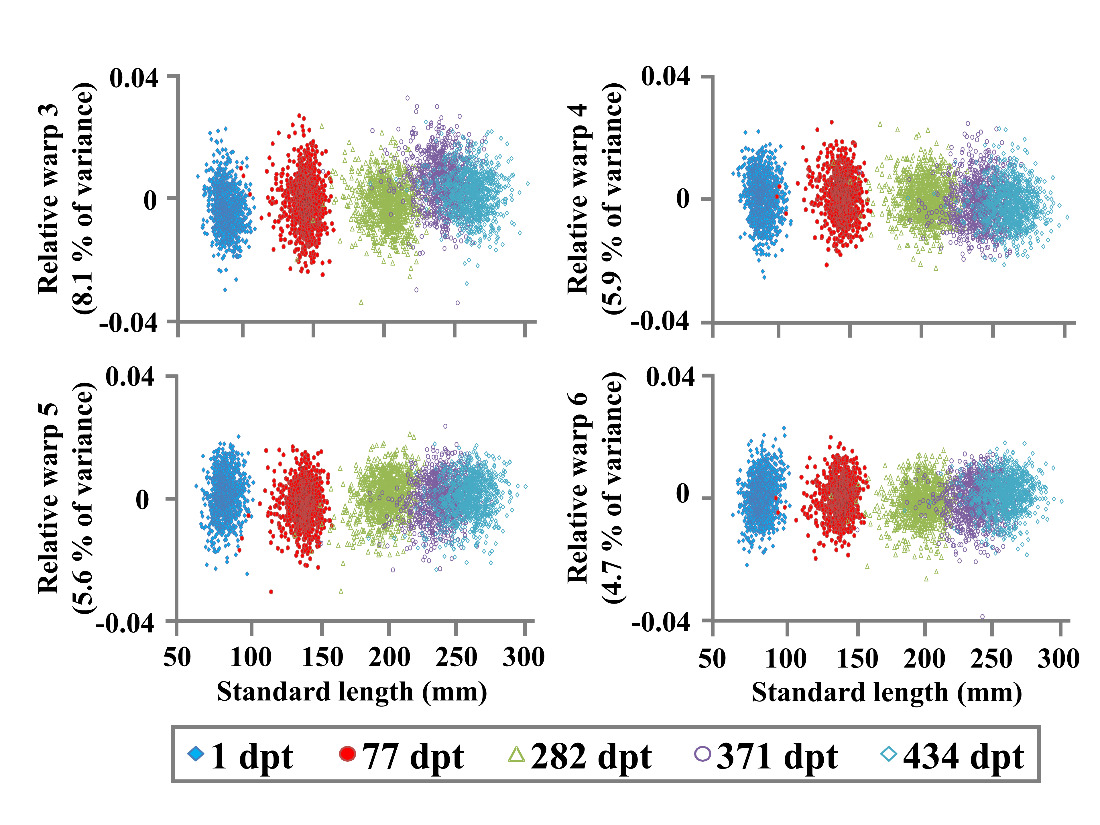


**Figure S1.** Relationship of the third, fourth, fifth and sixth relative warp (RW3-RW6) to standard length (SL) throughout the on-growing period.

**Table S1.** Estimated parameters of the fitted piece-wise regressions Y = b_0_ + b_1_·SL + b_2_·(SL − Lm)·(SL ≥ Lm), where Y is the score of Procrustes distance or the first Relative warp, SL is the standard length, b_0_ is the y-intercept, b_1_ is the slope of the relationship during the juvenile stage, b_2_ is the change in the juvenile slope (b_1_) that results in the slope of the relationship during the adult stage, and L_m_ is the SL at which slope changes (considered to denote the mean length-at-metamorphosis).SE, is Standard error

|  | **Procrustes distance (PD)** | **Relative warp 1 (RW1)** |
| --- | --- | --- |
| **b_0_(± SE)** | -0.0181 (±0.0) | -0.0589 (±0.0) |
| **b_1_(± SE)** | 0.0003 (±0.0) | 0.0004 (±0.0) |
| **b_2_(± SE)** | -0.0003 (±0.0) | -0.0004 (±0.0) |
| **L_m_(± SE)** | 192.7 (±1.9) | 202.0 (±1.7) |
| **R** | 0.832 | 0.853 |

**Table S2**. Mean Scores of the first and second relative warp (RW1, RW2) for the population percentiles (5-95%) in the size classes. The thirteenth class was pooled with the twelfth class, because it contained a single specimen.

| **Class** | **SL Range (mm)** | **Mean SL**  **(SD) (mm)** | **n** |  |  |  | **RW1** |  |  |  |  |  | **RW2** |  |  |
| --- | --- | --- | --- | --- | --- | --- | --- | --- | --- | --- | --- | --- | --- | --- | --- |
|  |  |  |  |  | **5%** | **25%** | **50%** | **75%** | **95%** |  | **5%** | **25%** | **50%** | **75%** | **95%** |
| 1 | 60.0-79.9 | 76.8 (3.0) | 194 |  | -0.046 | -0.034 | -0.029 | -0.023 | -0.018 |  | -0.003 | 0.009 | 0.016 | 0.021 | 0.030 |
| 2 | 80.0-99.9 | 87.4 (5.0) | 746 |  | -0.041 | -0.033 | -0.027 | -0.021 | -0.013 |  | -0.010 | 0.001 | 0.006 | 0.012 | 0.020 |
| 3 | 100.0-119.9 | 107.9 (7.6) | 35 |  | -0.038 | -0.029 | -0.022 | -0.015 | -0.010 |  | -0.010 | -0.004 | 0.001 | 0.005 | 0.016 |
| 4 | 120.0-139.9 | 134.6 (4.8) | 271 |  | -0.025 | -0.018 | -0.012 | -0.007 | 0.001 |  | -0.023 | -0.012 | -0.007 | -0.001 | 0.006 |
| 5 | 140.0-159.9 | 146.7 (4.4) | 669 |  | -0.024 | -0.015 | -0.009 | -0.004 | 0.004 |  | -0.023 | -0.014 | -0.008 | -0.003 | 0.005 |
| 6 | 160.0-179.9 | 171.4 (6.8) | 45 |  | -0.019 | -0.005 | 0.004 | 0.011 | 0.019 |  | -0.016 | 0.001 | 0.009 | 0.014 | 0.022 |
| 7 | 180.0-199.9 | 192.9 (5.2) | 326 |  | -0.004 | 0.007 | 0.014 | 0.020 | 0.028 |  | -0.009 | -0.001 | 0.005 | 0.010 | 0.016 |
| 8 | 200.0-219.9 | 209.0 (5.5) | 574 |  | -0.001 | 0.012 | 0.019 | 0.025 | 0.032 |  | -0.010 | -0.002 | 0.003 | 0.008 | 0.015 |
| 9 | 220.0-239.9 | 231.6 (6.1) | 436 |  | -0.012 | -0.001 | 0.007 | 0.014 | 0.027 |  | -0.014 | -0.005 | 0.001 | 0.006 | 0.013 |
| 10 | 240.0-259.9 | 250.0 (5.6) | 800 |  | -0.007 | 0.003 | 0.010 | 0.017 | 0.025 |  | -0.016 | -0.007 | -0.001 | 0.004 | 0.011 |
| 11 | 260.0-279.9 | 268.3 (5.4) | 612 |  | 0.001 | 0.009 | 0.015 | 0.022 | 0.031 |  | -0.017 | -0.008 | -0.002 | 0.002 | 0.009 |
| 12 | 280.0-299.9 | 285.3 (4.7) | 86 |  | 0.006 | 0.012 | 0.019 | 0.024 | 0.030 |  | -0.014 | -0.004 | 0.001 | 0.005 | 0.011 |
| 13 | 300.0-319.9 | 305.1 | 1 |  | 0.003 | 0.003 | 0.003 | 0.003 | 0.003 |  | 0.001 | 0.001 | 0.001 | 0.001 | 0.001 |

**Table S3**. Mean, standard deviation (SD), min and max values of individual trajectories. "b" is the slope, "a" the Y-intercept and r^2^ the coefficient of determination of the SL-RW1 and SL-PD linear regression, for each individual fish and the three first samples. pL-RW1, mean RW1 and for each individual fish at the three last samples. pL-PD, mean PD and for each individual fish at the three last samples. n gives the number of fish.

| **Character** | **Trajectory Parameter** | **Mean** | **SD** | **min** | **max** | **n** |
| --- | --- | --- | --- | --- | --- | --- |
| **PD** | **b** | 0.0053 | 0.0109 | -0.0287 | 0.0436 | 959 |
|  | **a** | 0.0003 | 0.0001 | 0.0000 | 0.0005 |  |
|  | **r^2^** | 0.93 | 0.11 | 0.13 | 1.00 |  |
|  | **pL-PD** | 0.0559 | 0.0062 | 0.0332 | 0.0078 |  |
| **RW1** | **b** | 0.0004 | 0.0001 | 0.0001 | 0.0008 |  |
|  | **a** | -0.0615 | 0.0145 | -0.1279 | -0.0179 |  |
|  | **r^2^** | 0.9436 | 0.07 | 0.34 | 1.00 |  |
|  | **pL-RW1** | 0.0126 | 0.0079 | -0.0152 | 0.0372 |  |
